# Intensive Care Unit Nurses in Iran: Occupational Cognitive Failures and Job Content

**DOI:** 10.3389/fpubh.2022.786470

**Published:** 2022-04-29

**Authors:** Fatemeh Mehri, Amin Babaei-pouya, Mansoureh Karimollahi

**Affiliations:** Department of Intensive Care Unit, Ardabil University of Medical Sciences, Ardabil, Iran

**Keywords:** job cognitive failures, job content, nurse, ICU, Iran

## Abstract

**Background:**

Nurses in intensive care units (ICU) are under a lot of stress because of special conditions caused by the work environment and the high level of knowledge and skills required to work in these units, which can lead to cognitive failures. This study aimed to investigate the relationship between occupational cognitive failures (OCF) and job content (JC) in nurses in the ICU of Ardabil hospitals in 2020.

**Methods:**

The present study was a descriptive-analytical cross-sectional study that was conducted in 2020. The study population included nurses working in the ICU of hospitals in Ardabil, from which 267 people who were eligible to enter the study were selected. OCF and JC questionnaires were used to collect data. Data were analyzed using SPSS software 23.

**Results:**

OCF with work records in the ICU, total work records, and work records in the COVID-19 and non-COVID-19 units are significantly associated. OCF was inversely related to the JC subscales of skill discretion and decision authority. And directly related to other subscales of JC.

**Conclusions:**

Develop job ability, reduce repetitive tasks, create diversity in work, create opportunities for creativity, have the authority and freedom to make decisions, facilitate work with new technologies, have enough time to do work, have a friendly work environment with colleagues, support by the supervisor, improving posture, especially for the upper body, feeling job security can help to reduce the cognitive failure of nurses.

## Background

Errors in the provision of health services are unsafe behavior and in some cases irreparable phenomenal. The nursing error means a failure to meet the standards of care that most of these errors occur when caring for patients so that annual nursing errors lead to increased length of hospital stay and increased medical costs (up to 9.14%) and even the death of thousands (1, 2). Annually, 44,000 to 98,000 people in the United States die due to medical errors, and deaths from preventable accidents in hospitals exceed the number of deaths attributed to vehicle accidents (3).

Nurses who are adapted to working conditions reduce errors and cognitive failures by focusing properly on their tasks. However, night work, long shifts, and unpredictable activities increase their fatigue can reduce their performance and physical capacity, and increase the likelihood of cognitive failures. Chronic drowsiness and fatigue are factors that affect the cognitive function of nurses and cause patient care to be dismissed and not done properly or be delayed (4). Cognitive failure is simple mistakes in daily activities, such as forgotten commitments and difficulty concentrating, that can lead to human error (5). Cognitive failures occur daily in the process of information processing in the stages of perception, memory, and motor actions, and human errors due to cognitive failures may occur in one of three stages of perception, memory, and motor actions (6). The results of several studies have shown that occupational cognitive failure (OCF) can lead to decreased safety in job performance (7, 8).

Cognitive failures as mind-related errors are related to the job content (JC) subscale. Job content refers to the evaluation of psychological and social stress factors including skill discretion, decision authority, psychological job demand, physical exertion, physical isometric loads, job insecurity, supervisor support, and coworker support (5). The existence of JC refers to factors that are controlled by the person in his job, such as performance, cognition, and independence, which are directly related to the job (5) and are strongly influenced by work stress. Among nurses, especially nurses in ICU, the increased workload is one of the most important causes of stress that increases cognitive failures, reduces the quality of care and patient safety (9). If people's abilities do not match their job conditions, it causes job stress and increases cognitive failures (4). Nurses who did not have good general health will not be able to provide better physical and mental care to patients, and this will increase mistakes and occupational accidents, which will ultimately affect the nurse and the patient (8).

According to our literature review, the relationship between OCF and JC of nurses has not been studied. In addition, since a significant number of people lose their lives due to medical errors (3) and one of the factors affecting medical errors is OCF and JC; Identifying the factors affecting OCF and JC in nurses can be an important step to reduce medical errors of nurses. Therefore, the present study was conducted to determine the relationship between OCF and JC in nurses of ICU of Ardabil educational and social security centers. The results of such studies can be of great help in improving and controlling the health status of patients and nursing staff.

## Methods

### Study Design and Participants

The present study was a descriptive-analytical cross-sectional study that was conducted in 2020. The study population included nurses working in intensive care units (ICU) of hospitals in Ardabil, from which all 267 people who were eligible to enter the study were selected. The study protocols were designed according to the Strengthening the Reporting of Observational Studies in Epidemiology (STROBE) statement, and it is approved by the Ardabil University of the medical sciences ethics committee.

### Inclusion and Exclusion Criteria

The inclusion criteria were the nurses with a bachelor's degree and higher, having the experience of working in the ICU for at least 6 months, having no history of severe mental illnesses, not receiving any treatment for serious diseases, and consent to participate. The exclusion criteria, having no will to continue the participation.

### Sample Size Calculation

According to enrolling all eligible individuals into the study, this is considered a consensus sampling.

### Data Collection

Data collection tools included a three-part questionnaire. The first part included a demographic information sheet (age, gender, and work records, level of education, marital status, work shift, employment status, and history of mental disorders), the second part included the OCF Questionnaire and the JC Questionnaire.

### OCF Questionnaire

The OCF Questionnaire was designed by Hassanzadeh Rangi et al. (10). This questionnaire has 30 questions and its answer range is of the 5-point Likert type, which is “I strongly disagree” with grade 1, “I disagree” with grade 2, “I have no opinion” grade 3, “I agree: with grade 4, and “I completely agree” with grade 5. Based on this questionnaire, the obtained scores are collected, and then the rate of OCF is judged based on the sum of scores. The minimum score is 30 and the maximum score is 150. A score between 30 and 60 indicates low cognitive failure. A score between 61 and 90 indicates moderate cognitive failure, and a score above 90 indicates high cognitive failure. Hassanzadeh et al. reported a content validity of 0.70 and its reliability by Cronbach's alpha method of 0.96 (10). in the study, Athar et al. this questionnaire by had used for hospital nurses (7).

### JC Questionnaire

JC Questionnaire has been developed by Kazarak et al. to measure JC (11). Factor validity of this questionnaire has been confirmed by the developers. Also, its reliability has been reported by Cronbach's alpha coefficient method for skill discretion 0.43, decision authority 0.64, psychological job demand 0.60, physical exertion 0.65, physical isometric loads 0.85, job insecurity 0.32, supervisor support 0.87, and coworker support 0.76 (12, 13).

The guide to the JC questionnaire includes the number of items, calculation formula, maximum and minimum, and average scores are given in Table 1.

**Table 1:** Guidance content questionnaire guide.

**Variable**	**Number of items**	**Formula**	**Minimum** **score**	**Maximum** **score**	**Average score** **(cut point)**
Skill discretion	6	[Q1 + Q3 + Q4 + Q5 + Q6 + (5-Q2)] ×2	12	48	30
Decision authority	3	[Q7 + Q9 + (5-Q8)] ×4	12	48	30
Psychological job demand	5	[(Q10 + Q11) ×3 + 15-(Q12 + Q13 + Q14)] ×2	12	48	30
Physical exertion	3	[Q15 + Q16 + Q17]	3	12	7.5
Physical isometric loads	2	[Q18 + Q19]	2	8	5
Job insecurity	3	[Q20 + Q22 + (5-Q21)]	3	12	7.5
Supervisor support	4	[Q23 + Q24 + Q25 + Q26]	4	16	10
Coworker support	4	[Q27 + Q28 + Q29 + Q30]	4	16	10

### Interviews and Data Collections

After explaining the objectives of the research and the demand for cooperation in researching the nurses in the first session, emphasis was placed on accuracy and honesty in completing the questionnaires and it was ensured that the information obtained would be completely confidential. According to the census sampling method, the questionnaires were provided to all nurses working in ICU with frequent visits in different shifts. Due to the busy work of the nurses in these units, to encourage them to cooperate and increase the accuracy in answering the questions, after distributing the questionnaire, they were asked to complete the questionnaires during their free time. The questionnaire was received in the same shift or at the time of re-visit.

### Statistical Analysis

Continuous variables were demonstrated as M ± SD, and categorical variables were described in count and percentage. Initial analyses did not show outliers, as assessed by a boxplot. The variables were confirmed for normal distribution with the Kolmogorov Smirnov test (*p* > 0.05); Also, the hypothesis of homogeneity of variances (sphericity hypothesis) was tested using the Mauchly test. The test results showed that the assumption of the equality of variance is established (*p* > 0.05). To evaluate the independence of categorical variables, a Chi-square test was used. The association between categorical and continuous variables was assessed using an independent samples t-test and one-way ANOVA. The correlation between continuous variables was investigated with Pearsons's coefficient. The statistical analysis was done using SPSS version 23 (SPSS Inc. Chicago, IL). A *p* < 0.05 was considered statistically significant in all tests.

## Results

The results showed that the mean age of the subjects was 34.6 ± 6.3 years. Most of the participants in this study were female (92.1%); The average work record was 10.45 ± 6.15 years. Other demographic information is present in Table 2.

**Table 2:** Demographic and basic characteristics of the participants.

**Variables**	**Categories**	**No**	**%**
Age (years)	Younger than 30	64	24
	30–39	136	50.9
	Older than 39	67	25.1
Gender	Female	246	92.1
	Male	21	7.7
Marital status	Married	210	78.7
	Single	57	21.3
Education	Bachelor's	247	92.5
	Master's degree	8	3
	PHD	12	4.5
Employment Status	Full-time	155	58.1
	Part-time	47	17.6
	Apprentice	37	13.8
	Contract based	28	10.4
Unit	Dialysis	28	10.5
	NICU	46	17.2
	ICU of emergency room	34	12.7
	ICU of COVID-19	93	34.8
	ICC of heart surgery	44	16.5
	CCU	22	8.2
Shift type	Fixed	28	10.5
	With rotations	239	89.5
History of non-severe mental illness	Yes	6	2.3
	No	261	97.7

As shown in Table 3, the M ± SD and range for scores of each subscale of JC and OCF are described.

**Table 3:** Mean and standard deviations of job content and OCF scores.

**Variable**		**Mean**	**SD**	**Minimum**	**Maximum**
Job content	Skill discretion	34.28	7.96	12	48
	Decision authority	34.61	5.4	12	48
	Psychological job demand	46.67	9.49	12	48
	Physical exertion	8.29	2.72	3	12
	Physical isometric loads	4.87	1.94	2	8
	Job insecurity	7.06	1.42	3	12
	Supervisor support	10.06	3.83	4	20
	Coworker support	10.49	3.75	4	16
OCF		83.41	19.96	30	150

Work records in the ICU, total work records, and work records in the COVID-19 and non-COVID-19 units are significantly associated with OCF and Skill discretion. In other words, with increasing work records, decision authority also increases.

Work in the COVID-19 ICU has a significant relationship with the psychological job demand, Physical exertion, and Physical isometric loads. In other words, the nurses who worked in the COVID-19 ICU experienced more psychological job demands, Physical exertion, and Physical isometric loads (Table 4).

**Table 4:** Association between JC, OCF, and characteristics of participants.

**Variable**	**Categories**	**OCF,** **Mean (SD)**	***P*** **-value**	**Skill discretion,** **Mean (SD)**	***P*** **-value**	**Decision authority,** **Mean (SD)**	***P*** **-value**	**Psychologicl job demand,** **Mean (SD)**	***P*** **-value**	**Physical exertion,** **Mean (SD)**	***P*** **-value**
Age (y)	Younger than 30	84.59 (19.29)	0.245	34.78 (7.55)	0.450	35.03 (3.98)	0.043*	47.84 (7.71)	0.319	8.26 (2.64)	0.487
	30–39	84.3 (19.3)		34.51 (7.92)		35.05 (5.22)		47.12 (10.36)		8.43 (2.66)	
	Older than 39	79.37 (21.56)		33.18 (8.498)		33.07 (6.47)		45.43 (9.28)		7.93 (2.95)	
Gender	female	82.83 (18.54)	0.345	33.71 (7.01)	0.548	34.61 (4.10)	0.996	46.88 (9.59)	0.767	8.22 (2.74)	0.436
	male	82.83 (18.63)		34.43 (8.22)		34.61 (5.70)		47.29 (9.21)		8.54 (2.64)	
Unit	COVID-19 ICU	86.95 (15.53)	0.022*	36.96 (6.79)	0.001*	35.23 (5.94)	0.144	48.53 (8.26)	0.041*	9.35 (2.82)	0.001*
	Non-COVID-19	81.49 (18.34)		32.81 (8.19)		34.29 (4.09)		46.13 (9.49)		7.68 (2.82)	
Work records (y)	Less than 7	79.1 (19.95)	0.001*	32.52 (8.16)	0.001*	33.46 (5.13)	0.013*	47.56 (8.65)	0.408	8.32 (2.72)	0.642
	7–15	83.29 (16.73)		33.87 (7.72)		34.59 (5.51)		47.23 (9.96)		8.41 (2.61)	
	More than 15	89.89 (17.34)		37.33 (7.39)		36.27 (5.49)		45.51 (9.80)		8.00 (2.96)	
Work recoeds in ICU (y)	Less than 5	80.11 (18.98)	0.002*	31.76 (7.42)	0.001*	34.51 (5.49)	0.781	47.64 (9.52)	0.233	8.11 (2.66)	0.260
	5 and more	87.23 (17.16)		36.68 (8.02)		34.71 (5.32)		46.26 (9.46)		8.48 (2.77)	
**Variable**	**Categories**	**Physical isometric loads**, **Mean (SD)**	***P*** **-value**	**Job insecurity**, **Mean (SD)**	***P*** **-value**	**Supervisor support**, **Mean (SD)**	***P*** **-value**	**Coworkers support**, **Mean (SD)**	***P*** **-value**		
Age (y)	Younger than 30	5.01 (1.94)	0.678	6.98 (1.28)	0.371	10.10 (3.92)	0.782	10.48 (3.67)	0.412		
	30–39	4.88 (2.00)		7.19 (1.58)		10.10 (3.69)		10.69 (3.60)			
	Older than 39	4.71 (1.84)		6.89 (1.19)		9.70 (4.03)		9.94 (4.10)			
Gender	female	4.88 (1.94)	0.881	6.99 (1.41)	0.124	9.89 (3.79)	0.186	10.34 (3.70)	0.236		
	male	4.88 (1.94)		7.34 (1.42)		10.71 (3.96)		11.03 (3.90)			
Unit	COVID-19 ICU	5.46 (1.87)	0.001*	7.22 (1.49)	0.205	10.65 (3.83)	0.084	10.78 (3.82)	0.363		
	Non-COVID-19	4.58 (1.87)		6.98 (1.37)		9.75 (3.81)		10.34 (3.72)			
Work records (y)	Less than 7	4.95 (1.89)	0.439	7.17 (1.39)	0.705	10.52 (3.96)	0.398	10.67 (3.72)	0.654		
	7–15	4.95 (2.05)		7.01 (1.51)		9.77 (3.64)		10.55 (3.56)			
	More than 15	4.58 (1.78)		7.00 (1.29)		9.96 (4.02)		10.10 (4.19)			
Work recoeds in ICU (y)	Less than 5	4.87 (1.96)	0.997	7.00 (1.42)	0.448	10.33 (3.87)	0.262	10.81 (3.64)	0.153		
	5 and more	4.87 (1.92)		7.14 (1.43)		9.78 (3.79)		10.15 (3.85)			

* *Statistically significant*.

*A one-way ANOVA or t-test was used as appropriate*.

According to the results of Table 5, OCF was inversely related to the JC subscales of skill discretion and decision authority, and directly related to other subscales of JC.

**Table 5:** Correlation coefficients with Pearsons' r between JC subscales and OCF.

**Variables**	**OCF**	**Skill** **discretion**	**Decision** **authority**	**Psychological** **job demand**	**Physical** **exertion**	**Physical** **isometric loads**	**Job** **insecurity**	**Supervisor** **support**	**Coworker** **support**
OCF	1	−0.597**	−0.217**	0.520**	0.737**	0.542**	0.478**	0.783**	0.713**
Skill discretion		1	0.319**	0.196**	0.387**	0.447**	0.200**	0.540**	0.381**
Decision authority			1	0.283**	0.077	0.072	0.043	0.071	0.035
Psychological job demand				1	0.331**	0.276**	0.243**	0.388**	0.428**
Physical exertion					1	0.443**	0.558**	0.588**	0.428**
Physical isometric loads						1	0.227**	0.336**	0.383**
Job insecurity							1	0.556**	0.293**
Supervisor support								1	0.830**
Coworker support									1

* *Significant at p < 0.05*.

** *Significant at p < 0.01*.

## Discussion

Most of the nurses working in the ICU had moderate levels of OCF. A review of literature in this area reveals different levels of occupational cognitive failure in nurses. The mean of cognitive failure in our study was higher than the mean reported in the study of Yousefzadeh et al. in nurses (1) and was consonant with the mean reported in the study of Mohammadi et al. in nurses. (14) and the study of Waltz et al. (15). Reisen (1997) has stated that job failure can be more due to failure in planning (mistakes) and implementation (cognitive failures). The work environment and the job of individuals, in general, can be the cause of occupational errors and cognitive failures in the individual (16), This is because the workload of nurses, especially nurses in the ICU, may cause problems and errors in the field of patient care because the ICU is a complex and stressful work environment (17), patients are more stressed (18), which may lead to occupational cognitive failures.

Nurses working in ICU in this study had high levels of skill discretion, decision authority, psychological job demand, physical exertion, supervisor support, and coworker support; on the other hand, the level of job insecurity and physical isometric loads was low.

According to the review of studies conducted in this regard, the results of the study of Gholami et al. (19) which was performed on 500 nurses of teaching hospitals in Hamadan, showed that the average component of freedom of decision is 64.67; psychological job demand 22.36; Social support 71.22; Job physical needs were 15.99 and job insecurity was 7.53, which was close to our study. Individuals' JC refers to factors that are self-controlled such as performance, cognition, independence, which are directly related to the individual's job (11) and affect the work stress of individuals. In general, job characteristics such as supervisor support for employees, job security, job independence, and the existence of a warm and friendly environment are among the factors that can affect the work aspects of people and lead to increased JC (20).

OCF was inversely related to the subscales of skill discretion and decision authority, and directly related to other subscales of JC. The skill discretion was directly related to all subscales of JC, and the decision authority was directly related only to the psychological job demand. The psychological job demand was directly related to all realms of JC. Also, the subscales of physical effort, isometric physical load, job insecurity, lack of supervisor support, and lack of coworker support were directly related to all subscales of JC except the decision authority subscale.

The results of the study of Hassanzadeh Rangi et al. (8) indicate a positive relationship between cognitive failures and workplace accidents which was consistent with the results of Park et al. (21), which showed a direct relationship between job stress and cognitive failure in nurses. However, it was not consistent with the results of the study of Barzideh et al. (22) which showed that there is no relationship between job stress and some job problems of nurses.

Work records in the ICU, and total work records, were significantly associated with OCF. In other words, nurses who worked in the ICU have experienced more job failures, and job failures also increase with increasing work records.

The study of Yousefzadeh et al. (1) showed that there was a significant correlation between cognitive failures with shift work, work records, and work departments (emergency, ICU); it also showed that there was no correlation between OCF and gender, the number of patients monitored, shift hours and rest hours. Moreover, the study of Mohammadi et al. (14) showed that there was no significant relationship between gender and job failures. However, the results of the study by Park et al. (21) showed that there was a significant relationship between nurses' gender and job failures.

It can be stated that the work records of the person in the COVID-19 ICU have caused stress and psychological pressure on medical team members, especially nurses, and dealing with critically ill patients also increases their fear, anxiety (22, 23).

Because cognitive failures as mind-related errors are directly related to job stressors, job stress is rooted in a person's inability to perform their duties (5) and is strongly influenced by the work environment.

The results showed that among the subsets of skill discretion, decision authority, psychological job demand, physical exertion, physical isometric loads were significantly associated with work records. These results were in line with the findings of Alacacioglu et al. and Kanai-Pak et al. (24, 25).

Working in the ICU requires that the staff, especially the nurses in these units, have the ability to use skills and have a great deal of decision-making power. As Apker et al. (26), ICU nurses have the ability to make quick and accurate decisions. In the ICU, teamwork is very important, when inexperienced nurses are placed next to professional nurses, they can increase their professional skills (26).

ICU is a complex and stressful work environment that is due to the critical nature of hospitalized patients, advanced devices and equipment used in the unit, and the need for speedy action of nurses in inpatient care. The nature of the ICU inevitably affects the cooperation and communication of nurses and causes the need for active participation in patient care, nurses 'respect for each other, and increasing nurses' trust and expertise (17). As the results of the present study showed, there was a significant relationship between work records, work in the ICU, and COVID-19 ICU with decision authority, physical exertion, psychological job demand, and other components.

Also, there was no significant relationship between head nurse support, coworker support, and job insecurity with any of the demographic characteristics of nurses. The results of the study by Yaser et al. (27) showed that there was no significant relationship between cognitive involvement with gender, education, age, work records, social responsibility, and type of unit in nurses. It can be stated that the majority of nurses studied were formally and contractually employed, so they were safe in their jobs. Accordingly, no significant relationship was observed between job insecurity and any of the demographic variables (27). The results indicate the fact that there is an inverse relationship between job security and stress and work pressures, especially work in the ICU. Accordingly, the attention of officials to the type of employment, employment conditions, and security that they provide for this important and sensitive segment of the health and medical system in terms of work, can provide the basis for providing better services.

Also, the result obtained in coworker support with demographic characteristics was inconsistent with the findings of Moore et al. (28). The results of Moore et al.'s study showed that there was a significant relationship between social interaction and cooperation with demographic characteristics (age, gender, education, work history) of nurses (28).

The results of the study showed that OCF was inversely related to skill discretion and decision authority, and directly related to psychological job demand, physical exertion, physical isometric loads, job insecurity, supervisor support, and coworker support.

The results suggest that paying attention to skill discretion, decision authority, psychological job demand, physical exertion, physical isometric loads, job insecurity, supervisor support, and coworker support can reduce OCF, and also consequently improve their productivity.

## Conclusion

The quality of nurses' activities is very important for patient safety, reducing the length of hospital stay and ultimately productivity. Human resource management should be done to reduce OCF.

To reduce the cognitive failure of nurses, the need to develop job ability, reduce repetitive tasks, create diversity in work, create opportunities for creativity, as well as have the authority and freedom to make decisions can help.

Other important things to reduce nurses' OCF are facilitating work with new technologies, having enough time to do work, having a friendly work environment with colleagues, supporting by supervisor and colleagues, improving posture, especially for the upper body, feeling job security.

For future studies, it is recommended to conduct a case study (using a control group) on the factors affecting OCF (Participant Characteristics, Professional ranks, Hospital level, Years of prior nursing experience...).

## Limitation

The limitations of this study include the limited statistical population of this study with nurses in intensive care units, which can be problematic in generalizing the results to other nurses. As well as the small number of male samples can affect the research results and should be considered in interpreting the findings.

One of the strengths of this study is the appropriate sample size and considering the dimensions of JC and their relationship with OCF.

## Data Availability Statement

The raw data supporting the conclusions of this article will be made available by the authors, without undue reservation.

## Ethics Statement

The studies involving human participants were reviewed and approved by Ethics Committee of Ardabil University of Medical Sciences. The patients/participants provided their written informed consent to participate in this study.

## Author Contributions

FM: data collection, analyzing data, and writing manuscript. MK and AB-p: research idea, research design, and writing manuscript. All authors contributed to the article and approved the submitted version.

## Funding

This project is funded by any official center and we declare that there is no actual or potential conflict of interest in relation to this article.

## Conflict of Interest

The authors declare that the research was conducted in the absence of any commercial or financial relationships that could be construed as a potential conflict of interest.

## Publisher's Note

All claims expressed in this article are solely those of the authors and do not necessarily represent those of their affiliated organizations, or those of the publisher, the editors and the reviewers. Any product that may be evaluated in this article, or claim that may be made by its manufacturer, is not guaranteed or endorsed by the publisher.
